# Roles of physical exercise in neurodegeneration: reversal of epigenetic clock

**DOI:** 10.1186/s40035-021-00254-1

**Published:** 2021-08-13

**Authors:** Miao Xu, JiaYi Zhu, Xian-Dong Liu, Ming-Ying Luo, Nan-Jie Xu

**Affiliations:** 1 Department of Anatomy, Histology and Embryology, Kunming Medical University, Kunming, 650500 China; 2 Collaborative Innovation Center for Brain Science, Department of Anatomy and Physiology, Shanghai Jiao Tong University School of Medicine, Shanghai, 200025 China; 3 Department of Neurology and Institute of Neurology, Rui Jin Hospital, Shanghai Jiao Tong University School of Medicine, Shanghai, 200025 China; 4 Shanghai Key Laboratory of Reproductive Medicine, Shanghai Jiao Tong University School of Medicine, Shanghai, 200025 China; 5 Key Laboratory of Cell Differentiation and Apoptosis of Chinese Ministry of Education, Shanghai Jiao Tong University School of Medicine, Shanghai, 200025 China; 6 Present Address: Department of Laboratory Medicine and Pathobiology, University of Toronto, Toronto, ON Canada

**Keywords:** Physical exercise, DNA methylation, Neural mechanism, Neurodegeneration, Motor deficits

## Abstract

The epigenetic clock is defined by the DNA methylation (DNAm) level and has been extensively applied to distinguish biological age from chronological age. Aging-related neurodegeneration is associated with epigenetic alteration, which determines the status of diseases. In recent years, extensive research has shown that physical exercise (PE) can affect the DNAm level, implying a reversal of the epigenetic clock in neurodegeneration. PE also regulates brain plasticity, neuroinflammation, and molecular signaling cascades associated with epigenetics. This review summarizes the effects of PE on neurodegenerative diseases via both general and disease-specific DNAm mechanisms, and discusses epigenetic modifications that alleviate the pathological symptoms of these diseases. This may lead to probing of the underpinnings of neurodegenerative disorders and provide valuable therapeutic references for cognitive and motor dysfunction.

## Background

The lack of physical exercise (PE) is a common phenomenon in modern society and has become a risk factor for many diseases, including cardiovascular diseases, metabolic dysfunctions, cancers, and neurodegenerative diseases [1–3]. In recent years, regular PE—whether aerobic exercise, anaerobic exercise, or resistance exercise—has been recommended as an essential component of healthy lifestyles. Appropriate exercise shapes the athletic figure and improves the body's basal metabolic rate [4]. PE also plays a vital role in brain health, especially in preventing and alleviating the decline of cognitive function as well as the occurrence of some neurodegenerative diseases [5, 6]. Due to the extensive impacts of brain health and the benefits of PE to physical fitness, systematic reviews and meta-analyses have emerged to sum up the possible connections, bringing us insightful conclusions with quantitative evidence [7, 8].

In healthy brain conditions, basic cognitive functions promote advanced brain competencies such as language skill, strategy learning, executive capability, and reasoning, which are essential for the development and progression of human society [9, 10]. Cognitive decline is often observed in rapidly aging populations [11], and in many cases, can progress to mild cognitive impairment or dementia with diagnosis of neurodegenerative diseases such as Alzheimer's disease (AD) and Parkinson’s disease (PD) [12, 13]. Therefore, topics around cognitive decline and neurodegenerative diseases during both normal and abnormal brain aging have become one of the leading issues on health. Several authoritative health-related research agencies including the National Institute on Aging in the U.S. Department of Health & Human Services have suggested the unequivocal effect of appropriate PE in improving cognition across populations (including children, adolescents, and older adults). The positive effects of regular, long-term physical activities and exercise interventions on cognition have also been reported in the literature [14, 15]. Since only limited therapies are available for cognitive impairment, exercise may serve as a promising non-pharmaceutical treatment [16].

**Fig. 1:**
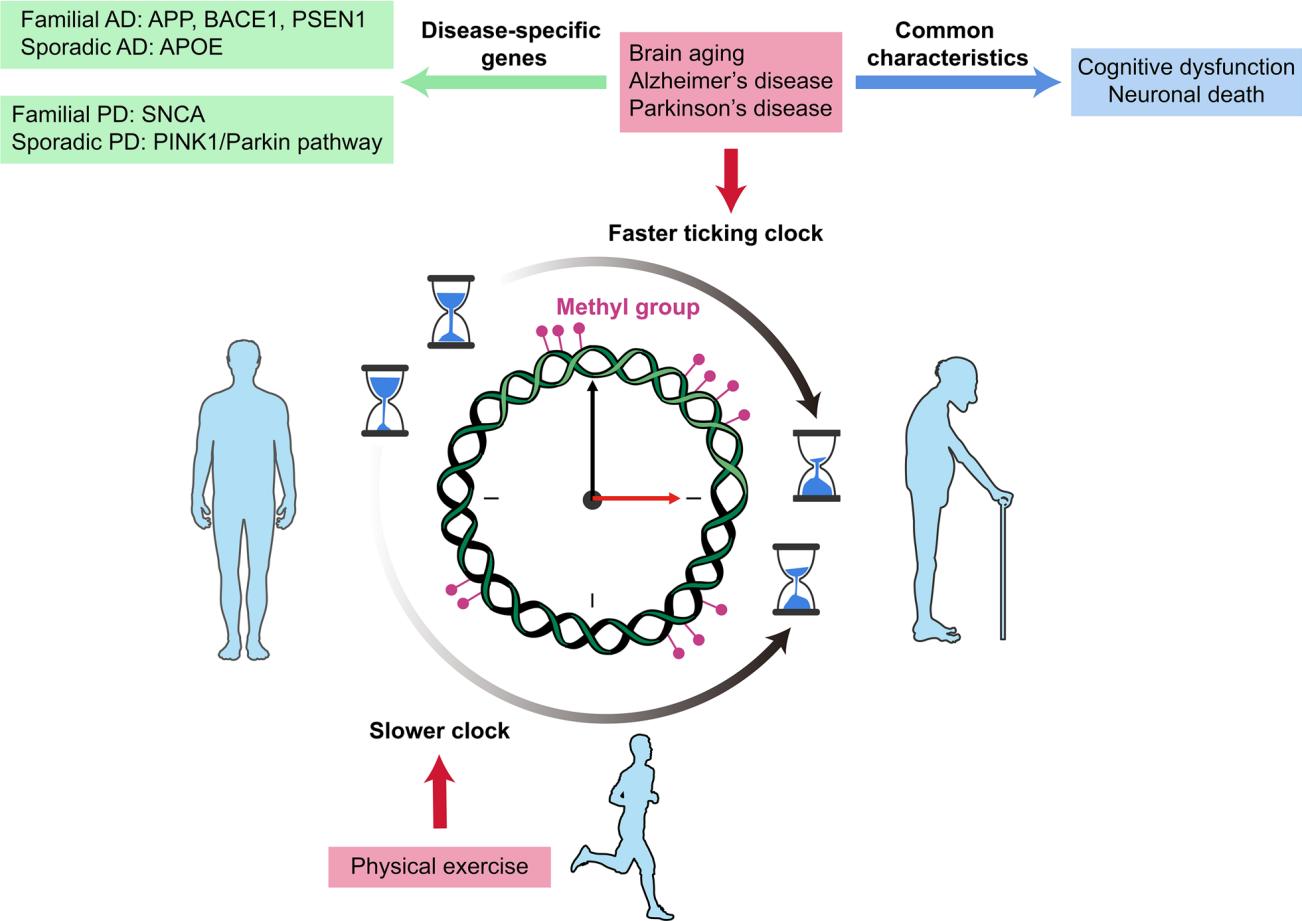
Physical exercise (PE) reverses the epigenetic clock in the aging brain and in neurodegenerative diseases. The epigenetic clock is associated with aged brain and neurodegenerative diseases, both of which show an accelerated epigenetic age. PE delays the clock by acting on their common pathologies (cognitive dysfunction and neuronal death) and disease-related genes underlying DNAm. AD: *APP*, *BACE1* and *PSEN1* in familial AD; *APOE* in sporadic AD. PD: *SNCA* in familial PD; *PINK1* and *Parkin* in sporadic PD

For the past few years, the process of brain aging, which is one of the risk factors for neurodegeneration, has been found to involve epigenetic mechanisms [17]. Epigenetics, by definition, refers to a set of heritable mechanisms and phenomena that determine cell phenotypes without changing the genome [18]. Epigenetic modifications such as abnormal DNA methylation (DNAm), microRNAs and histone modifications are closely associated with damage to brain health and neurodegenerative diseases [17]. DNAm is a fundamental epigenetic modification that coordinates gene expression, and its level has been regarded as a mark for age prediction [19]. As individuals age, the age-related changes are often linked to the fluctuating methylation levels of specific genes. The DNAm has been proposed as a potential muti-tissue estimator of biological age and the concept of epigenetic clock (i.e., DNAm clock) has been developed with a suitable regression model to systemically measure the biological age in all tissues and cell types except the sperm [20]. This tool has been extensively applied to distinguish between chronological age and biological age, as well as to estimate the corresponding health/disease status [21, 22]. While healthy individuals have almost identical chronological age and biological age (normal aging), patients with cancer and neurodegenerative diseases are biologically older (pathologic aging) and the offspring of centenarians are biologically younger (delayed aging) [21, 23, 24]. Therefore, the epigenetic clock is capable of assessing the state of aging among populations. Moreover, DNAm is associated with environmental and lifestyle factors, which have the capacity for regulating epigenetic variability in the brain [25, 26]. Given the effects of such factors as PE in slowing down the epigenetic age acceleration or even resetting the aging clock, the epigenetic clock has progressively become an exciting area of research [22]. In addition, these factors offer a possibility not only to delay disease progression and pathological aging, but also to promote rejuvenation. Therefore, PE has potentials to reverse the epigenetic clock against neurodegeneration (Fig. 1).

In this review, we summarize brain-specific, disease-related mechanisms involving DNAm (Table 1), through which PE reverses the epigenetic clock to ameliorate neurodegeneration in aging, AD, and PD (Table 2). We also integrate data from muscular-related molecule cascades in the periphery, which are directly induced by PE to affect the central nervous system (CNS). Furthermore, as a potential mediator of motor skills, DNAm can be modulated to improve the pathological symptoms of dyskinesia-related neurodegenerative diseases. The role of PE in neurodegeneration is further explored from the perspective of epigenetic-related mechanisms, and PE can be viewed as a potential rejuvenation therapy.

**Table 1:** Regulation of DNAm during neurodegeneration

References	Samples/subjects	Conditions	DNA methylation changes
Tan et al. [42]	5 ml blood samples of patients with PD	PD	CpG-2 of *SNCA* is hypomethylated
Cronk et al. [122]	C57Bl/6 J MeCP2-null mice	Rett syndrome	MeCP2 regulates microglial responsiveness and inflammatory gene transcription
McKinney et al. [83]	Postmortem orbital frontal cortices from 22 individuals (age > 60 years)	Brain aging	Ten of 26 CpG loci in *BDNF* are hypermethylated
Tarale et al. [45]	Human neuroblastoma cell line	PD	*PARK2* and *PINK1* are hypermethylated
Xie et al. [157]	Whole-blood samples from 506 aMCI patients	AD	Peripheral *BDNF* promoter methylation is elevated
Gontier et al. [133]	Aged (18-month-old) male C57BL/6 mice	Brain aging	Decreased Tet2 expression and 5-hmC levels in the aged hippocampus
Li et al. [8]	Individuals (Female: 47; Male: 54)	AD	Hypomethylated enhancers in the *DSCAML1* gene that targets BACE1
Balasubramanian et al. [119]	Adult male Wistar rats	Mild traumatic brain injury	Hypermethylation at *SOD2* promoter
Li et al. [134]	Aged APPswe/PSEN1 double-transgenic mice	AD	Decreased expression of Tet2

*5-hmC* 5-hydroxymethylcytosine, *AD* Alzheimer’s disease, *aMCI* amnestic mild cognitive impairment, *APP* amyloid precursor protein, *BACE1* β-site APP cleaving enzyme I, *BDNF* brain-derived neurotrophic factor, *MeCP2* methyl-CpG-binding protein 2, *PARK2* Parkin RBR E3 ubiquitin protein ligase, *PD* Parkinson’s disease, *PINK1* PTEN-induced putative kinase 1, *PSEN1* presenilin 1, *SOD2* superoxide dismutase 2, *Tet2* ten-eleven translocation methyl cytosine dioxygenases

**Table 2:** Physical exercise alters the levels of proteins related with neurodegeneration

References	Subjects	Intervention (duration/speed)	Observations
Ma et al. [129]	Adult male Wister rats with transient focal cerebral ischemia	Treadmill training: 3 days, 7 days, and 2 weeks, respectively, 12 m/min for 30 min each day, 5 days a week	Reduce the overexpression of TLR-2, TLR-4, NF-κB and MyD88 in rat brain tissues
Herring et al. [87]	210-day-old female TgCRND8 mice	Running wheels: 5 months	Reduce Aβ plaque burden and enhanced Aβ clearance
Tapia-Rojas et al. [88]	APPswe/PS1ΔE9 mice	Voluntary wheel running: 10 weeks	Decrease Aβ burden and Aβ oligomers in the hippocampus
Luo et al. [98]	Male Sprague–Dawley rats (16–18 months old)	Swimming exercise: 30 min per day, 5 days per week, 10 weeks	Upregulate the mRNA expressions of* Parkin*
Alkadhi and Dao [89]	7-week-old male Wistar rats: 2 weeks of Aβ infusion (250 pmol/day)	Treadmill exercise: 10–15 m/min, 4 weeks	Prevent the increase in the levels of APP, BACE1 and Aβ proteins in both the CA1 and DG areas
Daniele et al. [96]	Endurance athletes (mean age 41.4 ± 13.7 years)	Endurance athletes	Reduce the levels of total and oligomeric α-synuclein
Jessop and Toledo-Rodriguez [100]	C57BL/J6 male (3 months and 18 months old) mice	Running wheel: 4 weeks	Restore the age-related decrease in hippocampal *Tet1* and *Tet2* expression
Wu et al. [120]	Sporadic AD rat model	Swimming exercise: 4 weeks	Induce the DNA-binding activity of Nrf2 and expression of downstream antioxidant gene *Sod2* in the hippocampal CA1 region
El Hayek et al. [80]	Adult male C57BL/6 mice	Voluntary running wheel: 30 days	Increase *Bdnf* expression
Lourenco et al. [147]	AD model mice	Swimming exercise: 60 min per session, 5 days per week for 5 weeks	FNDC5/irisin mediates the protective effects of PE on synaptic plasticity and memory defects in AD
Just-Borras et al. [154]	ALS model mice	Treadmill training, swimming training: 30 min a day, 5 days a week, from 70 until 115 days of age	Maintain the BDNF/TrkB signaling at the neuromuscular junction

*Aβ* amyloid protein, *ALS* amyotrophic lateral sclerosis, *DG* dentate gyrus, *FNDC5* fibronectin type III domain-containing 5/irisin, *MyD88* myeloid differentiation 88, *NF-κB* nuclear factor-kappa B, *Nrf2* nuclear factor erythroid 2-related factor 2, *PE* physical exercise, *PS1* presenilin 1, *TLR-2* toll-like receptor 2, *TLR-4* toll-like receptor 4, *TrkB* tyrosine receptor kinase B

## Epigenetic regulation during aging and neurodegeneration

It is generally acknowledged that brain aging is distinct from neurodegenerative diseases—brain aging is a physiological condition whereas neurodegeneration is pathological. However, the two phenomena are interrelated since most aging adults would eventually encounter neurodegeneration, whose onset and progression are influenced by genetic and environmental factors. Therefore, aging is considered as a risk factor for the cognitive decline associated with neurodegenerative diseases whereas neurodegeneration is the manifestation of accelerated aging [27]. In normal brain aging, cognitive function gradually declines. In neurodegenerative disorders, the decrease in cognitive function is not the only symptom observed; major symptoms also include progressive damage in learning and memory, which could eventually lead to dementia. In addition, neuronal apoptosis is a natural physiological process that mediates controlled cell death. Although normal brain aging is regarded as an environmental factor contributing to apoptosis, the course of this process can be drastically accelerated in neurodegenerative diseases. Consequently, to some extent, the two conditions have similarities in symptoms and manifestations (e.g., memory and learning deficits) and the leading pathological features (e.g., neuronal death) [28].

As mentioned above, DNAm can not only predict the biological age due to its associations with brain aging, but is also involved in neurodegenerative diseases, implying its use to better visualize the pathogenesis. For example, in AD, the typical pathological features for diagnosis are senile plaque formation and neurofibrillary tangles (NFTs). The senile plaques are primarily formed from the deposition of beta amyloid (Aβ) proteins due to the abnormal shearing of amyloid precursor proteins (APPs) while the formation of NFT results from excessive phosphorylation and aggregation of tau proteins [29]. These neuropathological biomarkers of AD have been confirmed to be present in epigenetic age acceleration [30]. The accelerated aging caused by AD can be reflected by DNAm. For example, in a recent study, the DNAm age was shown to be 9 years older than the chronological age in an offspring of monozygotic triplets, who developed early-onset AD at age 50 [31]. Therefore, the accelerated epigenetic clock is associated with these neuropathological biomarkers, and may exacerbate the typical symptoms of declined global cognitive functioning, episodic memory, and working memory [30].

The biomarker- and symptom-related epigenetic age acceleration not only implies the close connection between AD progression and epigenetic clock, but also reveals changes in methylation levels of candidate genes. The pathogenic mutations of the *APP* gene, which are  the hereditary basis for familial AD, include considerable demethylation and increased expression [32]. As a membrane protein, APP concentrates in synapses of neurons and three enzymes (named α-, β-, and γ-secretases) participate in its proteolysis. In contrast to α-secretase, which is involved in healthy brain activity, successive cleavage of APP by β- and γ-secretases leads to the generation of neurotoxic Aβ [33]. The expression levels of these enzymes are controlled by methylation, which thereby regulates the disease progression. The overexpression of β-secretase, also known as β-site APP cleaving enzyme I (*BACE1*), is associated with hypomethylation of enhancer regions in the *DSCAML1* intron 3 that interact with the *BACE1* gene promoter in AD neurons [8]. Additionally, as part of the γ-secretase complex, presenilin 1 (*PSEN1*) is often observed with decreased methylation. In AD post-mortem human brains, the lower methylation of *PSEN1* compared to healthy controls contributes to higher expression of *PSEN1* that exacerbates neurodegeneration [34]. On the other hand, the most clinically relevant genetic risk factor for sporadic AD is *APOE* gene mutation [35]. Higher DNAm levels across the promoter region of the *APOE* gene may raise the risk of dementia and AD [36].

PD is another common neurodegenerative disease that has the fastest-growing prevalence, causing increased disability and death [37]. The main features of PD include the massive loss of DAergic neurons in the substantia nigra pars compact (SNpc), which causes blockage of dopaminergic afferent nerves in the basal ganglia and striatum [38, 39]. With the progressive deficiency of the DA system, the presence of cytoplasmic inclusion bodies (i.e., Lewy bodies, mainly composed of α-synuclein) in the residual neurons of the substantia nigra is considered as a prominent pathological change in PD. The accumulation of α-synuclein eventually leads to death and functional loss of DAergic neurons [40]. *SNCA* encodes for α-synuclein and is the first pathogenic gene discovered in familial PD. SNCA has been found to have dysregulated expression due to the abnormal methylation at its CpG sites [41, 42], which ultimately influences the content of Lewy bodies. Furthermore, additional PD-associated pathogenic genes can control the onset and progression of Parkinsonism. For example, PTEN-induced putative kinase 1 (*PINK1*) is a disease-causing gene that is involved in α-synuclein aggregation and regulation of dopaminergic neuronal homeostasis [43]. PINK1 is a crucial biomarker that links mitochondrial dysfunction with PD pathogenesis [44]. Hypermethylation of *PINK1* plays a crucial role in the etiology of early-onset PD [45]. *Parkin*, another PD-associated pathogenic gene located downstream of *PINK1*, functions in basal mitophagy by eliminating the damaged mitochondria to prevent inflammation and neurodegeneration [46]. A growing body of evidence implicates that mutations in *PINK1* are not only present in autosomal dominant, familial cases of PD, but also detected in sporadic PD patients [44, 47]. Given that only ~ 15% of PD patients have the monogenic origin and comprehensive epigenetic alterations are often observed in the genes described above, integration of DNAm data related to gene expression is essential for the identification of overlaps between sporadic and monogenic phenotypes [44].

Although the physiological changes and pathological causes of the above conditions are partially different, there are some common mechanisms in the epigenetics-related cerebral damage response. For example, a recent study identified 130 differentially expressed genes between AD cases and controls across four brain regions (the hippocampus, entorhinal cortex [EC], dorsolateral prefrontal cortex [PFC], and cerebellum), and found that the expression of these genes is associated with DNAm sites, which are overrepresented in AD genetic risk loci [48]. In addition, two loci (17q11.2 and 1p36.12) have been identified to be associated with epigenetic age acceleration of the PFC, and more importantly, the foundations of brain aging [49]. These results imply that DNAm is the underpinning of pathological and molecular changes in cognition-related brain regions. The concrete molecular mechanisms involve alterations of the DNAm profile in memory-related genes by DNA methyltransferase (DNMT) 3a2 [50], whose expression is reduced with aging in the hippocampus [51]. Furthermore, overexpression of neuronal DNMT3a2 leads to stable memory engrams and improves memory performance [50], implying a close relationship between DNAm and synaptic plasticity in neurodegeneration. The epigenetic states in neurodegeneration-affected patients and aging populations are both different from that in the general population and are associated with the methylation-related enzymes.

**Fig. 2:**
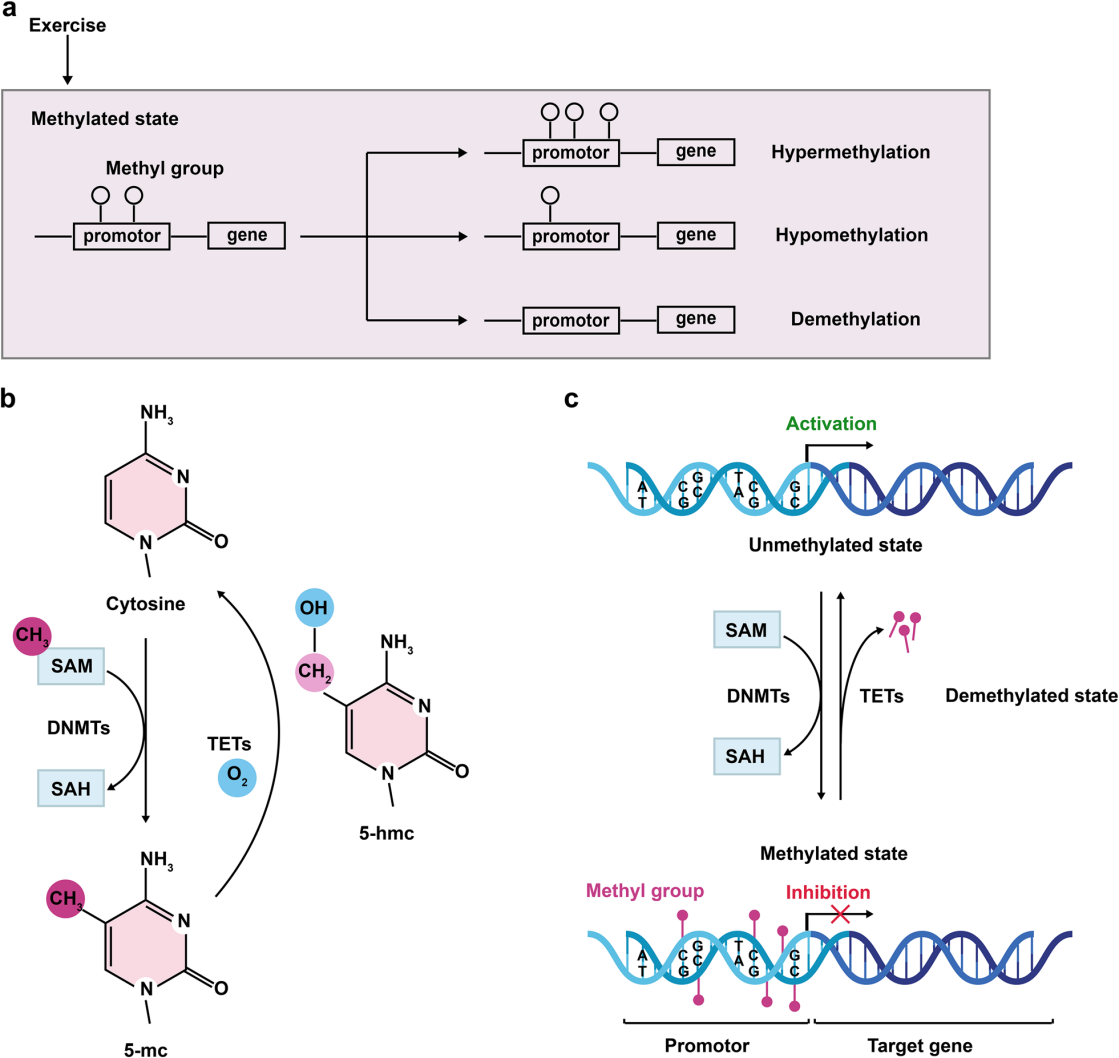
Exercise alters the state of DNAm. (**a**) Physical exercise changes the state of DNAm including hypermethylation, hypomethylation and demethylation to influence the expression of genes. (**b**, **c**) DNAm refers to the covalent bonding between a methyl group from S adenosyl methionine (SAM) and the carbon-5 position of cytosine in the genome CpG dinucleotide (catalyzed by DNMTs). 5-Hydroxymethylcytosine (5-hmC) is an intermediate during active DNA demethylation. The conversion of 5-mC to 5-hmC is mediated by TETs. DNAm generally leads to gene silencing, but the phenomenon is reversed in a demethylated state

Generally, the process of DNAm refers to the covalent bonding between the methyl donor S-adenosyl methionine (SAM) and the cytosine of the genome CpG dinucleotide, catalyzed by DNMTs, thereby regulating gene expression (Fig. 2) [52, 53]. DNMTs are a family of enzymes that include DNMT1, DNMT3a, and DNMT3b. The de novo methylases DNMT3a and DNMT3b transfer methyl groups to unmodified DNA strands, while the maintenance methylase DNMT1 is recruited to the newly synthesized daughter strand during DNA replication to promote a fully methylated state [54]. In certain cases of neurodegeneration, DNMTs show differential expression and enzymatic activities [55]. For example, current research suggests that the expression of DNMT1 decreases significantly from birth to senescence, and this is further supported by a comparison of hippocampal DNMT1 content between 3- and 20-month-old rats, and by data from aged 5×FAD mice [56, 57]. However, the level of DNMT1 has been reported to be significantly increased in 2-month-old AD transgenic mice [57]. The increase in DNMT1 during the initial stage of AD may be protective against the harmful effects that occur with AD. Thus, DNMT1 shows time-dependent level of expression in different life stages and diseases. In contrast, DNMT1 expression in the striatum increases with aging [58], indicating a regional specific pattern of DNMT1 expression.

The demethylation of 5-methylcytosine (5-mC) is mostly catalyzed by the ten-eleven translocation (TET) enzymes, including Tet1, Tet2, and Tet3. 5-Hydroxymethylcytosine (5-hmC) is the first oxidative product in the active demethylation of 5-mC, and is abundant in the CNS and functions to regulate neurodevelopment and synaptic function [59, 60]. The 5-hmC is now recognized as an important epigenetic marker in neurodegenerative diseases due to the significant differences in its level and TET expression between normal and pathological conditions [60]. In the hippocampus of AD patients, 5-mC and 5-hmC are robustly reduced, showing a significant negative correlation with amyloid plaque. Therefore, the progression of AD is accompanied by aberrant epigenetic signatures [61].

As a reader of DNAm, the enzyme methyl-CpG-binding protein 2 (MeCP2) can recognize the methyl-CpG-binding domain, and protect conversion of 5-mC to 5-hmC [62]. In addition, MeCP2 participates in neurodevelopment and maintains normal functions of the CNS by regulating DNAm dynamics, where both over- and under-expression of MeCP2 may lead to neuropsychiatric disorders [63]. Although *MeCP2* is a classical pathogenic gene of Rett syndrome, a rare neurodevelopmental disorder [64], studies have revealed that it is also dysregulated in AD and PD [65, 66]. Specifically, MeCP2 can bind to promoters at 5-mC regions to transcriptionally inhibit the expression of neurotrophin-like brain-derived neurotrophic factor (BDNF) [67]. A study has reported that APP/PS1 AD mice have decreased expression of MeCP2 and phosphorylated-cAMP response element-binding protein (p-CREB, an upstream factor of BDNF) in the hippocampus, suggesting the involvement of MeCP2/p-CREB in neurodegeneration by regulating the expression of neurotrophins [68].

## Exercise as an epigenetic protector against brain aging and neurodegeneration

As an external environmental modulator, PE has been confirmed to show beneficial effects against aging by increasing neuro-vascularization, neurogenesis, and neurotrophic factor synthesis [63, 69, 70]. Besides, as brain aging is also closely related to telomere length, which provides some indications of cell lifespan [71], a key goal of PE implementation is to increase telomerase activities and telomere lengths [72]. Telomerase reverse transcriptase (TERT), the catalytic subunit of telomerase, can influence the telomere-synthesizing DNA and maintain telomere stability [73]. However, TERT has been discovered to advance intrinsic epigenetic age acceleration in primary fibroblasts (although not in the brain), opposing its function in promoting proliferation [74].

Besides the example of telomeres, a recently published review summarizes a diverse set of circulating factors, such as BDNF, fibronectin type III domain-containing 5/irisin (FNDC5/irisin), and ketone bodies, that are affected by endurance exercise to counter age-related changes [75]. BDNF is required for almost all important cerebral functions, and plays multiple roles in the adult nervous system to support the survival, differentiation, and plasticity of existing neurons [76]. It also plays a regulatory role in neurodevelopment and synaptic transmission [76, 77]. Therefore, the progressive and dramatic decline of BDNF induced by brain aging is an important target for regulation. Since the first report of a positive correlation between exercise and BDNF mRNA level in rodents in 1995, a large number of studies have revealed the mechanisms underlying the effect of exercise on the epigenetics of BDNF [78–80], which controls BDNF expression and thereby regulates its functions in the brain [81, 82]. Specifically, there is a negative correlation between DNAm and BDNF expression, and aging induces increased methylation in *Bdnf* exons/promoters I, II, and IV in the orbital frontal cortex [83]. To a certain extent, exercise could positively contribute to the maintenance and improvement of brain health by modulating BDNF expression in the brain.

Both clinical and animal evidence has revealed that PE is an efficient therapeutic option for AD, with limited adverse effects reported in patients [84, 85]. PE alleviates the disease symptoms and delays the onset of cognitive deficits by directly counteracting Alzheimer-like pathologies [86, 87]. After 10 weeks of voluntary wheel training, the two major pathological biomarkers of AD, aggregated Aβ and phosphorylated Tau proteins, are significantly reduced in the hippocampus of a transgenic APP/PS1 mouse model. In addition, AD mice with running training demonstrate improved spatial memory and reduced neuron loss [88]. The expressions of *APP*, *BACE1*, and *PSEN1* in early-onset AD, or *APOE* in late-onset AD, are also regulated by exercise to varying degrees to alleviate the pathological symptoms of AD [89–91].

Aerobic exercise for 3 months in PD patients can effectively increase the evoked dopamine release in the caudate nucleus and the responsivity of the ventral striatum [92]. Moreover, regular PE leads to improvements in motor disorders (including tremor, cerebellar ataxia, and muscle rigidity) and non-motor performances (including autonomic dysfunction and cognitive deficits) in patients with mild to moderate PD [93–95]. Higher physical activity, rather than low levels of PE, can change the methylation status of *SNCA* and reduce the expression of both total and oligomeric α-synuclein [96]. PE also activates the PINK1/Parkin pathway to enhance mitophagy activity, which further promotes mitochondrial fitness and finally preserves cognitive function in the aged brain [97, 98].

Interestingly, researchers have found that PE is capable of controlling DNAm by regulating various enzymes to delay the processes of aging and neurodegeneration. For example, a single exercise session can decrease DNMT1 and DNMT3b levels in the hippocampus of young adult rats, but not in the aged group. The levels of the two enzymes remain unchanged in the aged rats even with a longer duration of an exercise protocol (i.e., chronic treadmill training) [56]. As a result, PE may not affect the expression and transcriptional activity of DNMTs in the aging brain but instead, may function as a neurodegenerative modulator that regulates the methylation of related genes (e.g., *BDNF*). In contrast to the regulation of DNMTs, the activity of DNA demethylation-related enzymes is directly regulated by PE in aged animals. A previous study showed that a 2-week, voluntary wheel training in sedentary rats increases the level of hippocampal Tet1 [99]. Additionally, 4-week PE counteracts the decreased Tet1 and Tet2 expression induced by aging, through enhancing the hippocampal 5-hmC content, thus promoting cognitive functions of aged mice [100]. In addition, PE can increase the level of activated MeCP2 that is closely related with BDNF expression. After 7 days of wheel running exercise, the phospho-MeCP2 level is upregulated, resulting in dissociation of MeCP2 from the *Bdnf* promoter, leading to *Bdnf* gene transcription [101].

## DNAm for neuroplasticity, neuronal loss and neurogenesis under exercise

The primary symptoms of neurodegenerative diseases are progressive cognitive decline and memory loss, which are mainly regulated by neuroplasticity, an adaptive ability to change brain structure and function in response to environmental stimulations [102]. It is well known that the hippocampus and the cortical region covering the PFC and the EC are commonly associated with learning and memory [103, 104]. Recent studies have shown that PE at a proper intensity can increase the volumes of the right and the left dorsolateral PFC, thereby interfering with the brain volume shrinkage that is commonly seen in aging populations, preventing the age-related deterioration of brain structures [105, 106]. Voluntary running can also enhance neurogenesis, which accelerates the recovery of synaptic plasticity from saturation to normality, and ultimately results in complete recovery of memory capacity [107]. It is evident that both MeCP2 and TETs serve as essential mediators of the hippocampus-dependent memory, such as regulations of LTP/LTD and excitatory synaptogenesis [108–110], and are positively modulated by exercise [101, 111, 112]. In conclusion, DNAm and demethylation are the keys to PE-related regulation of synaptic plasticity, promotion of memory encoding, and amelioration of cognitive defects.

Neurodegenerative diseases often eventually lead to loss of neuronal structures and functions. Neuronal death, whether apoptosis or necrosis, is caused by a variety of mechanisms related to DNAm. Studies on genetic and environmental factors in neurodegenerative diseases have shown that the imbalance between oxidative and antioxidant systems, caused by the increase in reactive oxygen species (ROS) and the deficiency of antioxidant capacity, is the main contributor to oxidative stress [113], which ultimately leads to neuronal death [113, 114]. Treadmill exercise can reverse the related neuronal loss by preventing oxidative damage of DNA [115]. In healthy cells, many important antioxidant-related factors and enzymes, such as nuclear factor erythroid 2-related factor 2 (Nrf2) and superoxide dismutase 2 (SOD2), are present and involved in defending against oxidative damage [116, 117]. In neurodegeneration, the decreased expression and function of such molecules is usually mediated by DNAm [118, 119]. In an AD rat model, exercise pretreatment significantly improves the protein level and DNA-binding activity of Nrf2, further promoting the expression of downstream antioxidant genes such as *SOD2*, leading to resistance to oxidative stress [120]. In an animal model of impaired redox homeostasis, occupancy of DNMT3b at SOD2 promoter causes hypermethylation at this site, and this hypermethylation is relieved after DNMT inhibition by a pan DNMT inhibitor, which relieves the oxidative damage and the deficits in learning and memory [119].

Neuroinflammation is a self-defensive response of the nervous system to harmful stimuli, and is persisting and hyperactive in neurodegenerative diseases throughout pathological progression. Apart from the role in restoring age-related deficits in cognition, PE can also suppress inappropriate neuroinflammation and its successive reactions by reducing the quantity of senescent microglia and increasing their phagocytic capacity [121]. The response of microglia to inflammatory stimuli is epigenetically regulated by MeCP2. MeCP2 deficiency promotes the transition of microglia into an active state and subsequently leads to microglia depletion as disease progresses [122]. Microglial activation triggers immune responses through the toll-like receptor 4 (TLR-4), a key regulator of the transcriptional factor nuclear factor-kappa B (NF-κB) in the innate immune system [123]. Subsequently, a wide variety of inflammatory cytokines are released. The activation of TLR-4 stimulates myeloid differentiation 88 (MyD88) and TNF receptor-associated factor 6 (TRAF6), which promote NF-κB release by phosphorylating the inhibitor of NF-κB (IκB) [124–127]. Interestingly, the activation of NF-κB decreases the expression of *TET* genes and thus induces mild aberration of methylation. Moreover, the enzymatic activities of DNMTs are upregulated as a result of the overproduction of nitric oxide (NO) in inflamed tissues. These two conditions synergistically cause abnormal methylation, which serves as an important mechanism underlying the involvement of chronic inflammation in neurodegenerative diseases [128]. Sufficient evidence has validated that PE can regulate the expression of TLR4, MyD88, TRAF6, and NF-κB in this signaling cascade [129–131]. Thus, the epigenetic regulation of neuroinflammatory proteins may underlie the effect of PE in decreasing neuroinflammation and its associated neuronal death.

PE alleviates the negative effects of DNAm associated with neurodegeneration and also regulates neuronal survival. DNMTs are involved in the regulation of neuronal survival and in the methylation processes in aging- and disease-related neurodegeneration. DNMT1 deficiency can counteract the age-related decline of cortical inhibitory interneurons in the cerebral cortex, as proved by *DNMT1*-deficient mice, which show increased interneuron survival and decreased age-related transcriptional changes [132]. This implies that DNMT1 is implicated in the reversal of the epigenetic clock by modulating age-related genes. While PE is ineffective in regulating DNMTs in older rats as depicted earlier [56], it is speculated that PE may indirectly affect these enzymes due to their critical role in neuronal survival and methylation. In addition to DNMTs, overexpression of Tet2 in the aged hippocampus can also counteract the decline in neurogenesis by increasing 5-hmC levels [133]. In middle-aged 2 × Tg-AD mice, over-expression of Tet2 in the dentate gyrus (DG) improves memory impairment and reduces amyloid burden [134], suggesting a neuroprotective role of Tet2 in neuronal survival. However, a recent study has found that the increased Tet2 modifies the enhancer sites in PD neurons to give rise to a loss of nigral dopaminergic neurons, while Tet2 depletion reverses the phenomenon [135]. Thus, the effect and activity of Tet2 may be regionally or neuronally specific.

In addition to maintaining neuronal viability, PE also promotes neurogenesis for neuronal renewal. Radial neural stem/precursor cells (rNSPCs) are quiescent under normal conditions and are regarded as a latent reservoir for neurons that participate in neurogenesis [136, 137]. Studies have found that exercise could transit rNSPCs from quiescence to an active state and induce their entry into the cell cycle [138]. Exercise can increase neurogenesis and ameliorate cognitive decline through a liver-to-brain axis, as an increase in liver-derived circulating blood factor glycosylphosphatidylinositol-specific phospholipase D1 (Gpld1) and several activated coagulation and complement signaling cascades have been identified in the brain after exercise [70]. Our previous work has revealed the activation of rNSPCs in the DG region, which is mediated by long-term excitation of hippocampal dentate granule cells upon 30 consecutive days of voluntary running in mice. By downregulating the ephrin-B–EphB transcellular signaling, this exercise training promotes the transition of quiescent rNSPCs and their acquisition of neuronal fate [137]. When subjected to certain stimuli, these activated rNSPCs may undergo asymmetrical division, giving rise to rapidly proliferating amplifying neural progenitors, which immediately differentiate into neurons [139]. The intrinsic state of rNSPC, whether quiescent or active, can be determined by the relative level of cellular ROS content. Physical activity drives ROS fluctuation to prepare rNSPCs to enter the cell cycle. Subsequently, the cells with lower ROS content exit the quiescent state and transform to a state with proliferative and differentiative activity [140].

PE not only promotes NSPC differentiation into neurons, but also maintains the homeostasis of the stem cell pool [137, 141]. The rNSPC pool declines with time after initial activation, which, together with brain aging, leads to impairment of hippocampal neurogenesis [139, 142]. In the aging hippocampus, oscillations of glucocorticoid hormones (GCs) prevent the activation of NSPCs induced by the glucocorticoid receptor (GR)–GC pathway, regulate their proliferation, and preserve a quiescent NSPC pool. This effect is mediated by the GC oscillation-induced changes in methylation of specific gene promoters associated with cell cycle regulation, and the maintenance of stable DNMT expression [143]. Interestingly, only oscillatory GCs (but not continuous GCs) show sensitivity to cell cycle entry, proliferation, and cell cycle exit [143]. Although evidence has shown that PE can induce the elevation of basal GC levels [144], the potential of PE in promoting GC oscillations remains unknown.

## The epigenetic clock of the muscle-brain crosstalk

PE induces muscle contraction and elicits muscle-brain signal transmission, during which myokines are secreted from muscle cells as an important element of the endocrine loop to stabilize the muscle–brain connection [63].

As a newly discovered myokine peptide, the soluble irisin is a cleaved and secreted fragment of FNDC5, and serves as a messenger molecule that is transmitted from muscles to various body tissues during exercise. Irisin also regulates neurogenesis, behavior, and metabolism in the brain [145, 146]. Peripheral overexpression of FNDC5/irisin in the livers of mice rescues the AD-associated memory defects, indicating that peripheral irisin may reach the brain and potentially mediate the neuroprotective actions of PE [147]. Remarkably, DNAm in the CpG island of the *FNDC5* promoter regulates *FNDC5* mRNA expression in the human liver through the binding of the transcription factor GR complex  to the targeted *FNDC5* gene [148]. In AD patients and mice, the levels of FNDC5/irisin in the brain and cerebrospinal fluid are significantly reduced, leading to impaired synaptic plasticity and memory. Yet, these alterations are reversed by exercise [147]. During PE, peripheral delivery of FNDC5 boosts blood irisin and further induces the expression of neuroprotective genes including *Bdnf* in the hippocampus [149]. Therefore, FNDC5/irisin serves as a crucial mediator of the beneficial effects of PE on cognition, suggesting that both peripheral and CNS FNDC5/irisin  are potential targets for AD treatment [147]. Tracing back to its sources, *FNDC5* gene expression is regulated by a transcriptional co-activator, peroxisome proliferator-activated receptor gamma coactivator 1-alpha (PGC-1α). PGC-1α is induced in muscles by exercise and mediates many biological processes related to energy metabolism [145]. In human skeletal muscles, PE reduces the methylation level of the *PGC-1α* promoter in an intensity-dependent manner, which leads to an increase in mRNA level [150]. PGC-1α deficiency causes neurodegenerative damage and reduces the expression of neuronal FNDC5 and BDNF in the brain [149, 151]. Therefore, the PGC-1α–FNDC5–BDNF pathway is an important regulator in the muscle-brain crosstalk.

In addition to the neuronal origin of BDNF that is regulated by PGC-1α-FNDC5/Irisin [149], BDNF is also directly secreted as a myokine from muscle cells during PE in response to muscle contraction. A meta-analysis has reported that the peripheral BDNF level in older adults (aged ≥ 60 years) can be increased by a variety of PE, thereby exerting neuroprotective effects on the brain [152]. The muscle-derived BDNF is capable of remodeling neuromuscular synapses and connections between motor neurons and muscles [153]. In the context of neurodegeneration, a lack of BDNF would exacerbate the age-related decline of muscle mass and function, as well as the loss of neuromuscular junctions. Exercise intervention can reverse many physiological changes associated with BDNF decline in peripheral blood, thereby supporting motoneuron survival in older adults [152, 154]. Besides the alterations in intracerebral BDNF, the differential methylation of the *Bdnf* gene in blood is considered as a biomarker of AD [155]. Both the transition from healthy state to dementia and the development from aMCI to AD are accompanied by increased methylation at the *Bdnf* promoter in blood [155–157], suggesting that the expression of peripheral BDNF changes with progression of pathology.

## Epigenetic modulation to reverse motor deficits

While it is tempting to conclude that the connection between DNAm and PE largely lies in the alteration of DNAm, this is not the case. In reality, DNAm can influence motor abilities and modulate behaviors reversely to sustain normal movements. For example, deletion of DNMT3a in AgRP neurons can reduce voluntary exercise behavior by disrupting the expression of genes that are characteristic of AgRP neurons in the arcuate nucleus of the hypothalamus [158].

The normal locomotor activity is balanced by DNMT3a levels, where both deficiency and overexpression of DNMT3a may lead to negative consequences. Knockout of DNMT3a interferes with the development of motor neurons and causes defects in neuralization [159], whereas overexpression of DNMT3a induces apoptosis of motor neurons in the mouse spinal cord and human motor cortex, leading to neurodegeneration [55]. On the contrary, excessive expression of DNMT3a2 and its partner DNMT3L driven by the dopamine transporter promotor activates the nigrostriatal pathway to improve locomotor function and spontaneous activity of DA neurons [160]. Interestingly, ablation of muscle-specific DNMT3a only influences the expression of genes involved in muscle development but does not alter the exercise capacity [161]. This suggests that DNMT3a regulates the movement ability through motor-related neurons in the brain rather than through mature skeletal muscles. Therefore, similar to the regulation of DNMTs by PE, the reverse modulation of motor deficits also has a clear regional specificity.

In dopaminergic neurons derived from induced pluripotent stem cells from patients with parkin (*PARK2*) gene mutations, there is hypomethylation of individual CpG sites at the catechol-O-methyltransferase (COMT) gene promotor, and increased expression of COMT [162]. In addition, overexpression of COMT in dopaminergic neurons of the substantia nigra induces impaired synaptic dopamine transmission and produces cataleptic behaviors that are associated with impaired motor coordination in the initial PD stages. Therefore, COMT upregulation may be regarded as an initial dysregulation in PD [162]. In addition, downregulation of Tet2 in the SNpc can reverse the PD-induced motor deficits and dopaminergic neuronal injury [163].

Huntington's disease (HD) is another common neurodegenerative condition with motor deficits, which is associated with mutations of the expanded cytosine-adenine-guanine (CAG) repeats in the huntingtin (*HTT*) gene. Similar to AD and PD, HD also exhibits an accelerated epigenetic age. Only the CpGs located in proximity (within 2 kb) to the CAG expansion in exon 1 of *HTT* are significantly hypermethylated in HD, which exhibit positive correlations with severe motor progression in patients [164, 165]. Amyotrophic lateral sclerosis (ALS) is the most common neurodegenerative disease of the motor neuron system, which is generally caused by mutations in the chromosome 9 open reading frame (*C9orf72*) gene [166]. Hexanucleotide repeat expansion of *C9orf72* causes downstream molecular aberrations and leads to overt cellular toxicity [167]. *C9orf72* promoter hypermethylation induces transcriptional silencing of *C9orf72,* which could maintain motor neuronal survival and serve as an endogenous protective regulator of neuropathology [167, 168]. In conclusion, most neurodegenerative diseases are related to epigenetic changes (i.e., hypomethylation and hypermethylation) in pathogenesis. Epigenetic alterations could be a therapeutic target to ameliorate the disease-related symptoms including movement disorders.

## Considerations and prospects

It is well known that PE is beneficial for brain health, yet it is influenced by a complex web of factors, which poses challenges for researchers to pinpoint the most appropriate exercise proposal. For example, PE influences neural mechanisms in an intensity-dependent manner. The cortical and subcortical brain regions respond more strongly to low-intensity exercise (ratings of perceived exertion [RPE] 6–12 on the Borg Scale) than to high-intensity exercise. Moreover, the cerebellum is only activated by low-intensity exercise. In contrast, the cognition-related area PFC shows reduced activation in low-intensity exercise, which is further exacerbated in high-intensity exercise (RPE 13–17) [169] as it may lead to body instability and stress responses [169, 170]. The benefit of low-intensity exercise has also been demonstrated in other performances. Compared with a running training at 20 m/min, the ischemia rats trained at 8 m/min show better spatial memory, enhanced hippocampal dendritic complexity, and increased BDNF level [170]. Yet, the positive effects of high-intensity training should not be undervalued, as high-intensity interval training shows greater enhancement of motor cortex plasticity compared with moderate exercise [171]. In addition to intensity, there are also concerns regarding the safety of training and the selection of exercise mode and duration. Although the use of PE as a therapeutic treatment remains partially controversial, there is no doubt a positive effect of exercise in neurodegeneration. PE can interfere with the disease-specific mechanisms, including abnormal aggregation of Aβ and APP in AD and functional loss of DA neurons in PD, which are correlated with DNAm in the epigenetic clock. The improvement of neurodegeneration by PE intervention is a time-dependent process, which alters the gene methylation differently at distinct disease stages. Besides, the expression of BDNF, which depends on the methylation level, is essential to most aspects of brain health and has regulatory effects on diverse cerebral functions both in the CNS and in the muscle-brain crosstalk. In DNAm and demethylation, the expression of essential enzymes—DNMTs and TETs—is time-specific, brain regional specific, and neuronal specific. Therefore, special attention should be paid to distinguish and refine the brain areas and neuronal cell types, as well as the stages of aging and diseases, when studying the effects of PE on these enzymes. Furthermore, current research has suggested that exercise only affects DNMT levels in young adult mice but not in aging brains. This conclusion should be made with caution due to the regional specific expression of DNMTs and the limited number of studies available. Future studies are needed to test the effect of PE in the aging brain and investigate the action specificity of DNMTs. In addition, mechanisms underlying the alterations of related genes and proteins caused by DNAm are potential targets for PE treatment, which is of significance for both healthy individuals and patients with brain dysfunctions. Future studies should also examine other genes that may be regulated by DNAm, in order to identify novel targets of PE.

## Conclusions

PE has a high potential for clinical improvement of symptoms of neurodegenerative diseases and for the reversal of aging, and prevention of age-related diseases. However, due to individual differences, the intensity of exercise has to be adjusted according to individual’s physical qualifications, health status, and disease development. Limitations presented in literature and on clinical applications indicate that a future direction of research is to explore the well-defined boundaries of PE to guide optimal exercise prescription and maximize the safety of PE treatments.

## Data Availability

Not applicable.

## Abbreviations

5-hmC: 5-Hydroxymethylcytosine
5-mC: 5-Methylcytosine
Aβ: Amyloid-β
AD: Alzheimer's disease
AE: Aerobic exercise
APP: Amyloid precursor protein
BACE1: β-Site APP cleaving enzyme I
BDNF: Brain-derived neurotrophic factor
CNS: Central nervous system
COMT: Catechol-O-methyltransferase
DG: Dentate gyrus
DNAm: DNA methylation
DNMT: DNA methyltransferase
EC: Entorhinal cortex
Gpld1: Glycosylphosphatidylinositol (GPI)-specific phospholipase D1
GR: Glucocorticoid receptor
HD: Huntington's disease
MyD88: Myeloid differentiation 88
NF-κB: Nuclear factor-kappa B
NFT: Neurofibrillary tangles
Nrf2: Nuclear factor erythroid 2-related factor 2
NSPC: Neural stem/progenitor cell
PD: Parkinson’s disease
PE: Physical exercise
PFC: Prefrontal cortex
ROS: Reactive oxygen species
SAM: S adenosyl methionine
SNpc: Substantia nigra pars compacta
SOD2: Superoxide dismutase 2
TERT: Telomerase reverse transcriptase gene
TETs: Ten-eleven translocation methylcytosine dioxygenases
TLR-4: Toll-like receptor 4
TRAF6: TNF receptor-associated factor 6
